# *Campylobacter* infection and household factors are associated with childhood growth in urban Bangladesh: An analysis of the MAL-ED study

**DOI:** 10.1371/journal.pntd.0008328

**Published:** 2020-05-14

**Authors:** J. Johanna Sanchez, Md. Ashraful Alam, Christopher B. Stride, Md. Ahshanul Haque, Subhasish Das, Mustafa Mahfuz, Daniel E. Roth, Peter D. Sly, Kurt Z. Long, Tahmeed Ahmed

**Affiliations:** 1 Children’s Health Research Centre, Faculty of Medicine, University of Queensland, Brisbane, Queensland, Australia; 2 Nutrition and Clinical Services Division, International Centre for Diarrheal Disease Research, Bangladesh (icddr,b), Mohakhali, Dhaka, Bangladesh; 3 The Institute of Work Psychology, University of Sheffield, Sheffield, United Kingdom; 4 Department of Pediatrics, Hospital for Sick Children and University of Toronto, Toronto, Ontario, Canada; 5 Department of Epidemiology and Public Health, Swiss Tropical and Public Health Institute, Basel, Switzerland; Mohammed Bin Rashid University of Medicine and Health Sciences, UNITED ARAB EMIRATES

## Abstract

The dual burden of enteric infection and childhood malnutrition continues to be a global health concern and a leading cause of morbidity and death among children. *Campylobacter* infection, in particular, is highly prevalent in low- and middle-income countries, including Bangladesh. We examined longitudinal data to evaluate the trajectories of change in child growth, and to identify associations with *Campylobacter* infection and household factors. The study analyzed data from 265 children participating in the MAL-ED Study in Mirpur, Bangladesh. We applied latent growth curve modelling to evaluate the trajectories of change in children’s height, as measured by length-for-age z-score (LAZ), from age 0–24 months. Asymptomatic and symptomatic *Campylobacter* infections were included as 3- and 6-month lagged time-varying covariates, while household risk factors were included as time-invariant covariates. Maternal height and birth order were positively associated with LAZ at birth. An inverse association was found between increasing age and LAZ. *Campylobacter* infection prevalence increased with age, with over 70% of children 18–24 months of age testing positive for infection. In the final model, *Campylobacter* infection in the preceding 3-month interval was negatively associated with LAZ at 12, 15, and 18 months of age; similarly, infection in the preceding 6-month interval was negatively associated with LAZ at 15, 18, and 21 months of age. Duration of antibiotic use and access to treated drinking water were negatively associated with *Campylobacter* infection, with the strength of the latter effect increasing with children’s age. *Campylobacter* infection had a significant negative effect on child’s growth and this effect was most powerful between 12 and 21 months. The treatment of drinking water and increased antibiotic use have a positive indirect effect on linear child growth trajectory, acting via their association with *Campylobacter* infection.

## Author summary

*Campylobacter* infection is a gastrointestinal bacterial infection that is widespread, particularly in low- and middle-income countries. Children in poor living conditions are particularly at risk for infection and are often also suffering from poor linear growth. We conducted an analysis of data collected from children in urban Bangladesh who participated in the MAL-ED study, a project that examined the effect of gastrointestinal infection on growth. We used innovative analytic methods to examine the linear growth of the children from 0 to 24 months of age and whether *Campylobacter* infection as well as household factors were associated with growth. We found that the height of the mother and the birth order of the child had a positive relationship with child’s length at birth. *Campylobacter* infection was found to have a negative impact on linear growth in children between 12–21 months of age. A longer duration of antibiotic use in the children, as well as the treatment of drinking water in the household had a negative relationship with *Campylobacter* infection, thus indirectly had a positive effect on the children’s growth. The negative relationship between *Campylobacter* and growth highlights the importance of reducing infection and addressing associated factors, such as water quality.

## Introduction

Growth impairment in children continues to be a major global health concern, particularly in low- and middle-income countries (LMICs). Linear growth in early childhood is an important indicator of nutritional status and is associated with both short- and long-term negative consequences, such as mortality, chronic disease, neurodevelopmental outcomes, and poor economic performance in adulthood [1–4]. Linear growth impairment, expressed as low height/length for age, is partly a consequence of fetal growth restriction, preterm birth, intergenerational effects. It also is a result of low household socioeconomic status which leads to inadequate dietary intake and inadequate sanitation, water quality and poor hygienic conditions; all of which contribute to greater exposure of children to infections and clinical disease [2,5–7]. Enteric infection has been recognized as an important cause of child growth impairment since enteric infection rates are negatively associated with child growth, particularly in LMICs [8]. This may be due to decreased nutrient absorption, inflammatory responses, reduced appetite, changing feeding practices, and the high prevalence of frequent episodes of infection in these settings [5,9–11].

The ‘Etiology, Risk Factors, Interactions of Enteric Infections and Malnutrition, and the Consequences for Child Health and Development Study’ (MAL-ED) was a multi-centre longitudinal research project designed to examine the effect of enteric infection and risk factors on child growth. The study screened stools collected from both diarrheal episodes and non-diarrheal stools for a wide variety of pathogens to estimate the pathogen-specific burden of diarrhea in children 0–24 months of age. *Campylobacter* was identified as one of the most prevalent enteric pathogens among children who participated in the study and was negatively associated with growth attainment at 24 months of age [2,12]. *Campylobacter* species are bacterial pathogens transmitted to humans through food, contact with animals, water sources and through person-to-person transmission via fecal oral route or fomites [13]. Asymptomatic infections in MAL-ED study participants were negatively associated with linear growth, a finding consistent with other studies that suggest that asymptomatic enteric infections may play a larger role in growth faltering and malnutrition than has previously been appreciated [2,14,15]. A cohort study in Peru also identified a marginal association between both symptomatic and asymptomatic *Campylobacter* infection and linear growth impairment in the 9 months following infection [16,17]. Asymptomatic *Campylobacter* infection is associated with prolonged excretion in many cases and is said to be an opportunistic infection as it is more common among children who are malnourished [18]. In a recent MAL-ED analysis, Rouhani *et al* reported that *Campylobacter* infection was associated with disruptions in the gut microbiota, which may explain the effects of asymptomatic infection on linear growth[19].

Bangladesh has one of the highest burden of growth impairment in the world despite important declines in recent decades [20]. Dhaka, Bangladesh’s capital city, has become one of the world’s most densely populated urban areas, and urbanization continues to increase (12). Urban settlements disproportionately attract economically disadvantaged rural population, resulting in crowded housing provision with poor environmental conditions [20]. This presents a public health challenge, as the households and their children are more vulnerable to disease. Studying the factors associated with enteric infection and childhood linear growth in such an urban context is crucial in addressing this public health problem.

Previous studies have explored the drivers of growth impairment in children; including household-level factors such as water, sanitation, and hygiene (WASH), child characteristics, and maternal factors. However; few have linked these factors to both specific enteric pathogen data and longitudinal growth trajectories. Latent growth curve modelling, a less commonly used approach outside of the social sciences, allows the application of many predictors and determines which exert important effects on the rate of change in growth, including both time-varying and time-invariant predictors or covariates. In addition, this method allows the opportunity to also explore the indirect effect of household factors on LAZ via infection. The application of such a method could contribute additional detail to the growing body of information on *Campylobacter* and growth.

Therefore, using the latent growth curve modelling approach, this study aims to investigate, for children in the Dhaka, Bangladesh MAL-ED study site: 1) whether these household-level factors and *Campylobacter* infection are associated with changes in LAZ across the first 24 months of life; 2) whether the effect of *Campylobacter* infection on changes in LAZ is consistent across the first 24 months of a child’ age or whether children are more susceptible to this effect at certain ages; and, 3) whether the effects of household-level factors on LAZ operated directly, or operated indirectly via their association with *Campylobacter* infection.

## Methods

### Study sample

A total of 265 children were enrolled within 17 days of birth between 2010 and 2012 from the Bauniabadh area of Mirpur, one of the 21 administrative units of Dhaka. Inclusion criteria included a birthweight or enrolment weight of greater than 1500g, a mother aged at least 16 years, singletons, and no other siblings being enrolled. Children with congenital or severe disease were excluded from the study [2,12,21].

### Study data collection

Length and weight measures of children were taken every month following their enrollment, using standard scales (Seca GmbH & Co. KG., Hamburg, Germany). LAZ scores (length-for-age z-score), were then calculated by mapping the individual child length, standardized by age and size, onto the WHO reference population distribution [22]. Non-diarrheal stool samples were collected from children every month for the first 12 months of age, and every 3 months from children between 12 and 24 months of age. Diarrheal stool samples were collected during a diarrheal episode or up to one day after. Diarrhea was defined as the maternal report of three or more loose stools in 24 hours [12]. Our analysis included the enteropathogen data collected from the non-diarrheal stool samples. Laboratory analyses of all stool samples were undertaken at a laboratory at icddr,b in Dhaka, Bangladesh, in accordance with a standardized microbiology protocol. *Campylobacter* was identified using Enzyme Linked Immunosorbent Assay (ELISA) ProSpecT kits, in accordance with manufacturers procedures [12,23]. [12,23]. The ProSpecT Campylobacter microplate assay (Alexon-Trend) detects Campylobacter Specific Antigen (SA), a surface antigen. The ELISA method was the preferred analysis method as it was found to have higher sensitivity than culture [24]. *Campylobacter* species was the most prevalent enteropathogen in the MAL-ED Bangladesh site, with prevalence increasing with age, from 18% children having positive stool samples at 3 months of age to 69% children at 24 months of age (S1 Table).

Household sanitation, and hygiene (WASH) characteristics were collected from caregiver interviews at study baseline. These included water source, treatment of drinking water, toilet facility type, whether toilets were shared, handwashing behaviours, and the presence of a refrigerator. Caregiver data, such as years of education, age, and anthropometric measures, including height, were also collected at study baseline.

### Statistical analysis

Latent growth curve modelling (LGCM) was used to assess the overall shape of growth in LAZ, variation in this pattern between the children studied, and antecedents of that variation. LGCM, a form of longitudinal analysis that utilizes a structural equation modelling (SEM) framework, models change in an outcome through estimating latent variables for the intercept (starting point) and slope (trajectory) of change, using the observed values of the variable across time as indicators of these underlying ‘true’ latent variables. Potential predictors of the intercept and trajectory of change can then be included in the model, both as subject-level predictors (i.e., time invariant constructs) and time-specific effects. [25]

Given the exploratory nature of part of our analyses—we did not have a clear hypothesis regarding the shape of the change in LAZ and the temporal pattern of the relationship between *Campylobacter* infection and LAZ—we split the data into random halves, with one half of the data used to build our model, and the other used for testing the fit of the resulting model and the statistical significance of its parameters. This was necessary to ensure robustness when evaluating our final model: a model built and then tested on the same subjects would naturally be likely to achieve a higher level of fit than when tested on any other random sample from the population to which we wish to generalize.

Model building was initiated with the development of an ‘unconditional’ growth curve model, which identified the trajectory of LAZ without the inclusion of any covariates. In this model LAZ measurements at 3-month intervals (0, 3, 6, 9, 12, 15, 18, 21, 24 months) were used as indicators of the intercept and slope latent variables, with the intercept mean describing the population-average baseline LAZ, the (linear) slope mean representing the population average rate of change of LAZ over time (per 3 month period), and intercept and slope variances respectively describing how starting level and change in LAZ varied amongst our subjects. To examine the improvement offered by a curvilinear model, an additional latent variable representing quadratic change was added to the model. We used the model chi-square statistics, Comparative Fit Index (CFI), Tucker-Lewis Index (TLI), Root Mean Square Error Approximation (RMSEA) and the Standardized Root Mean Square Residual (SRMR) Root to assess model fit [26]. A CFI value of above 0.95, a TLI value above 0.95, an SRMR value of below 0.08, and an RMSEA below 0.06 are all indicative of a good model fit [27]. Akaike Information Criterion (AIC) and Bayesian Information Criterion (BIC) were also used to compare between models when assessing relative model fit.

*Campylobacter* infection, defined as a positive stool sample collected from children regardless of clinical status, was then included as a time-varying covariate of LAZ. As such, our 3-monthly observations of LAZ (as opposed to the latent variables representing intercept and slope for change in LAZ) were regressed upon our dichotomous measurement of *Campylobacter* presence/absence. Given that the effect of infection on linear growth is delayed by several months, we lagged the effect *of* Campylobacter infection on LAZ by 3 and 6 month periods (e.g. LAZ at 9 months was regressed upon *Campylobacter* infection at both 3 and 6 months; LAZ at 12 months was regressed upon *Campylobacter* infection at 6 and 9 months, etc) [28–30]. We explored temporal variation in the effect of *Campylobacter* infection on LAZ by first fitting a model in which the 3-month lag and 6-month lag effects were both free to vary across all time points; having examined the pattern of coefficients from this model, we then fitted a series of simpler models based on these patterns, with fixings in place to model the effect of *Campylobacter* infection on LAZ being stronger/weaker at particular periods of the child’s development.

Finally, the subject-level demographic, family, and household factors collected at study baseline were considered as potential predictors of the latent variables for intercept, slope and quadratic change (i.e., having a direct effect upon LAZ), and of *Campylobacter* infection at each time point (therefore enabling an indirect effect on LAZ via *Campylobacter* infection). Specific household factors considered were exposure to treated drinking water, type of toilet facility, shared toilet facilities, and presence of a refrigerator; demographic factors were sex of the child, birth order, and maternal education. Duration of antibiotic use and presence of exclusive breastfeeding were also included as time-varying covariates in the model given their importance in infection and growth [8]. We examined whether the effects of these demographic and household variables on *Campylobacter* infection were consistent across time or showed variation by first fitting a model in which they were free to vary, and then checking whether there was loss in model fit when applying temporal fixings to these relationships: specifically, whether they were each constant across time, or whether they would have an increasingly (or decreasingly) important effect on *Campylobacter* infection throughout the 24 months. Again, models were compared using the fit indices listed above. The model emerging from this building process was then tested on the other half of the data, to obtain a robust evaluation of model fit and the statistical significance of model parameters. Mplus version 8.0 software was used for all LGCM modelling [31]. Models were estimated using Monte Carlo integration.

### Ethics statement

The MAL-ED study was approved in Bangladesh by the icddr,b review committee. Informed consent was obtained from the parent or guardian of every participating child in the study. The project was granted an Exemption to Ethics Review at the University of Queensland, as a secondary data analysis. An ethics application was also submitted to the regional ethics board in Switzerland (Ethikkommission Nordwest- und Zentralschweiz” (*EKNZ*)), on behalf of the Swiss Tropical and Public Health Institute, which granted a Declaration of No Objection.

## Results

Characteristics of study participants included in the final model are show in Table 1.

**Table 1:** Characteristics of study participants in the final model.

**Child Characteristics (N = 265)**	**N**	**%**
Sex (female)	136	51.3
Birth order		
1	108	40.8
2	94	35.5
3+	63	23.77
	**Mean**	**SD**
Duration of antibiotic use (mean days)	109.6	56.7
Duration of exclusive breastfeeding (mean days)	98.64	57.4
**Household Characteristic (N = 242)**	**N**	**%**
Drinking water source		
Piped into dwelling	197	81.4
Piped to yard/plot, public tap/stand pipe	45	18.6
Households treating water	146	60.3
Improved toilet facility	184	76.0
Shared toilet facility	201	83.1
Presence of refrigerator	68	28.1
**Maternal Characteristics (N = 265)**	**Mean**	**SD**
Maternal education (years)	4.54	3.2
Maternal height (cm)	148.9	5.2

Our exploratory (model building) analyses on one half of the data involved the development of an unconditional model, which estimated the starting point and change in the outcome variable LAZ without the inclusion of any covariates. The initial model included only the intercept and slope as latent variables, with their means and variances freely estimated (AIC = 1292.534, BIC = 1335.78, CFI = 0.918, TLI = 0.924, SRMR = 0.092, RMSEA = 0.162). To investigate whether a curvilinear trajectory of LAZ offered a better fit to the data, a quadratic term was added to the model, resulting in a substantial improvement in all model fit indices (AIC = 1237.20, BIC = 1291.98, CFI = 0.95, TLI = 0.95, SRMR = 0.078, RMSEA = 0.128).

We then explored temporal variation in the relationship between *Campylobacter* infection and LAZ. A model in which these regression coefficients were free to differ across time suggested that the effect was strongest for children over 12 months old but diminished as the children approached 24 months of age. Therefore, we compared the free model to one in which the effects on LAZ at 12, 15, 18, and 21 months were fixed to be equal. This was done separately for the 3- and 6-month lag *Campylobacter* infection to LAZ paths to account for potential difference in time length effect. We then separately fixed the effects on LAZ at 3, 6, 9 and 24 months to be equal. This latter model (i.e. the one with two sets of fixed equal paths (12–21 month and 3–9, 24 month) provided the best fit.

Finally, we explored the direct and indirect effect of household factors. The latent variables for the intercept, slope and quadratic term, and *Campylobacter* infection at each time point were regressed on child, maternal and household characteristics. Maternal age was not found to improve the model and therefore completely removed. Paths from toilet facility, antibiotic use, and exclusive breastfeeding to LAZ intercept, slope and quadratic terms, were likewise removed. The relationships with *Campylobacter* infection were fixed across all intervals as per exploratory results, instead of being allowed to vary freely. LAZ at each time point was regressed on the time-lagged *Campylobacter* infection variables, again with the equality restrictions across time suggested by our exploratory analysis in place.

**Fig 1:**
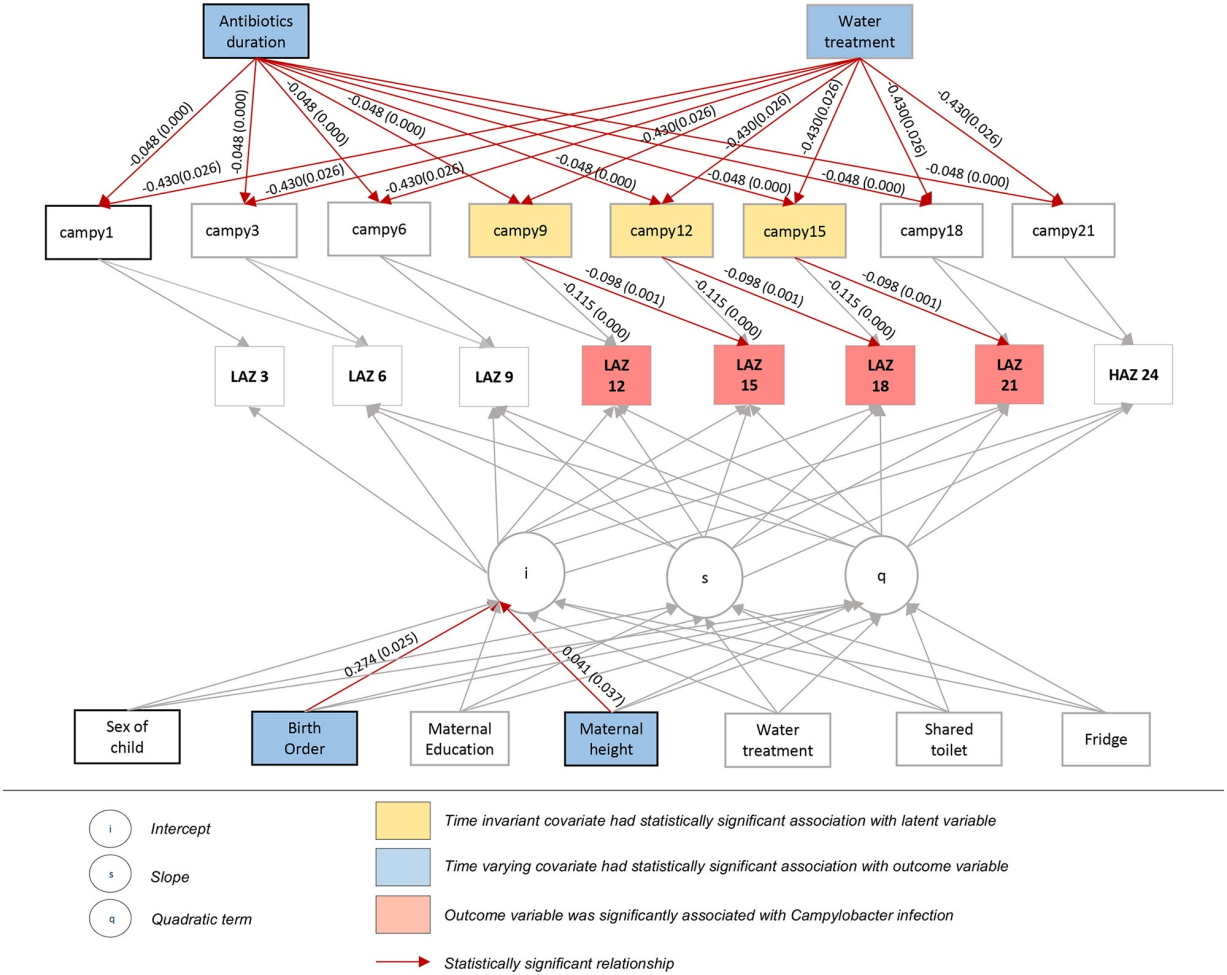
Final latent growth curve model: Final model results are presented in this figure. *Campylobacter* infection in the previous 3- and 6-month interval was negatively associated with LAZ at 12 to 21 months. Birth order and maternal height were significantly associated with baseline LAZ. Antibiotic use and treatment of water was negatively associated with *Campylobacter infection*.

We then tested our proposed final LGCM on the other half of the data. This model explained 69% of the variance in LAZ observed scores, through both the growth curve structure and the time-varying covariates of *Campylobacter* infection and breast-feeding; likewise our subject level predictors explained 17% of the variance in the intercept factor (i.e. initial level) of LAZ, 15% of the variance in the linear slope factor for change in LAZ, and 13% of the variance in the quadratic slope factor for LAZ. The percentage of within-subjects variance in LAZ uniquely explained by *Campylobacter* was 7.1%. Model results are presented in Table 2 and Fig 1.

**Table 2:** Final LGCM results for LAZ change over time and association with Campylobacter infection and household-level factors.

Dependent variable	Independent variable	Unstandardized Path Estimate	Standard error	P-value
*Growth factors associations with household level risk factors*				
LAZ intercept	Sex of child (1 = male, 2 = female)	0.144	0.195	0.459
	Birth order (1,2,3+)	0.274	0.122	0.025
	Maternal education (years)	0.011	0.037	0.772
	Maternal height (cm)	0.041	0.01	0.037
	Water treated (y/n)	-0.007	0.203	0.973
	Shared toilet (y/n)	0.044	0.275	0.872
	Presence of refrigerator (y/n)	0.294	0.231	0.203
LAZ slope	Sex of child (1 = male, 2 = female)	0.114	0.067	0.091
	Birth order (1,2,3+)	-0.052	0.042	0.215
	Maternal education (years)	0.016	0.013	0.204
	Maternal height (cm)	0.001	0.007	0.998
	Water treated (y/n)	0.109	0.070	0.116
	Shared toilet (y/n)	-0.003	0.094	0.974
	Presence of refrigerator (y/n)	0.009	0.079	0.905
*Effect of Campylobacter infection and exclusive breastfeeding on LAZ intervals*				
LAZ 6 months	Campylobacter—3 months	0.006	0.033	0.853
	Exclusive breastfeeding—3 months (y/n)	0.080	0.043	0.065
	Exclusive breastfeeding—6 months (y/n)	0.079	0.135	0.558
LAZ 9 months	Campylobacter—3 months	-0.018	0.036	0.614
	Campylobacter—6 months	0.006	0.033	0.853
	Exclusive breastfeeding—6 months (y/n)	0.000	0.115	0.999
LAZ 12 months	Campylobacter—6 months	-0.018	0.036	0.592
	Campylobacter—9 months	-0.115	0.030	<0.0005
LAZ 15 months	Campylobacter—9 months	-0.098	0.030	0.001
	Campylobacter—12 months	-0.115	0.030	<0.0005
LAZ 18 months	Campylobacter—12 months	-0.098	0.030	0.001
	Campylobacter—15 months	-0.115	0.030	<0.0005
LAZ 21 months	Campylobacter—15 months	-0.098	0.030	0.001
	Campylobacter—18 months	0.006	0.033	0.872
LAZ 24 months	Campylobacter—18 months	-0.018	0.036	0.592
	Campylobacter—21 months	0.006	0.033	0.872
*Effect of household factors on Campylobacter Infection*				
**Time-fixed covariates**				
Campylobacter 1-24mos	Sex of child (1 = male, 2 = female)	0.055	0.189	0.770
	Maternal education (years)	-0.011	0.030	0.722
	Water treated (y/n)	-0.430	0.193	0.026
	Improved toilet facility (y/n)	0.141	0.208	0.499
	Shared toilet (y/n)	0.135	0.240	0.574
	Antibiotic duration (days)	-0.048	0.009	0.000
**Time-Varying covariates**				
Campylobacter 3mos	Exclusive breastfeeding—3 months (days)	-0.647	0.602	0.283
Campylobacter 6mos	Exclusive breastfeeding—3 months (y/n)	-0.294	0.500	0.557
	Exclusive breastfeeding—6 months (y/n)	1.607	0.860	0.062
Campylobacter 9mos	Exclusive breastfeeding—6 months (y/n)	-0.402	0.843	0.634

**Fig 2:**
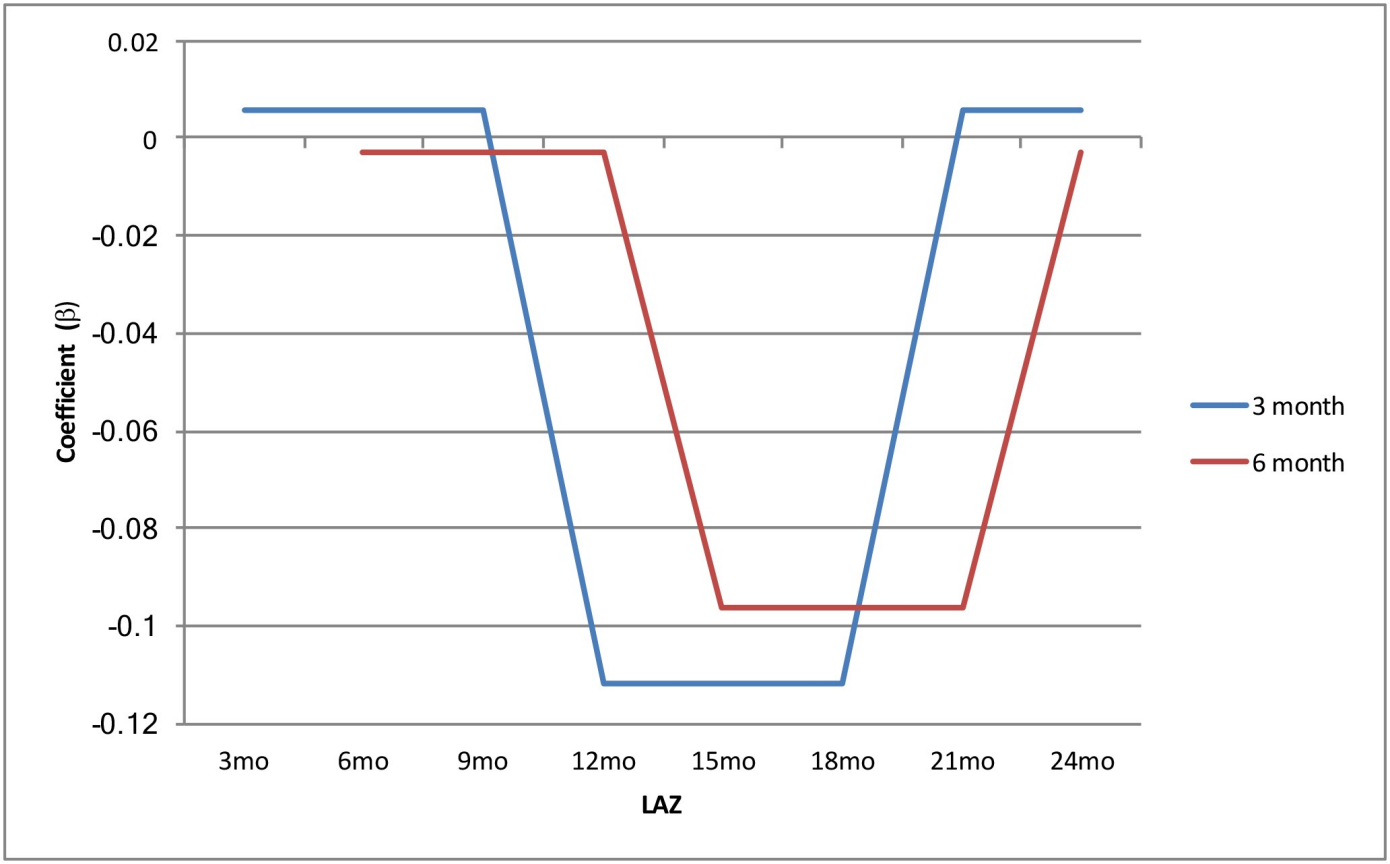
Relationship between *Campylobacter* infection across time intervals: *Campylobacter* infection in the previous 3-month interval (blue) was negatively associated with LAZ at 12, 15, and 18 months, while Infection in the previous 6-month interval (red) was negatively associated with LAZ at 15, 18, and 21 months.

*Campylobacter* infection in the previous 3-month interval was negatively associated with LAZ at 12, 15, and 18 months (b = -0.115, p = <0.001) (Figs 1 and 2). Infection in the previous 6-month interval was negatively associated with LAZ at 15, 18, and 21 months, (b = -0.098, p = 0.001) (Figs 1 and 2).

Of the demographic and household predictors, maternal height (b = 0.042, p = 0.033) was unsurprisingly positively associated with the intercept factor (i.e. the initial level of LAZ). Birth order was also significantly associated with initial LAZ (b = 0.274, p = 0.025). Increased duration of antibiotic use was negatively associated with *Campylobacter* infection (b = -0.048, p = <0.001). Water treatment was negatively associated with *Campylobacter* infection, with this effect equally fixed across time (b = -0.430, p = 0.026). Type of toilet facility, having a shared facility, and the presence of a refrigerator were not associated with *Campylobacter* infection at any time point.

Significant indirect effects, both operating via *Campylobacter* infection between the 12th and 21st months of the child’s life, were found between water treatment and LAZ (total indirect effect for the 3 and 6 month *Campylobacter* infection-LAZ lags, indirect effect = 0.092, SE = 0.046, p = 0.047) and between antibiotic use and LAZ (total indirect effect for the 3 and 6 month *Campylobacter* infection-LAZ lags, indirect effect = 0.010, SE = 0.003, p = 0.001). Indirect effects of water treatment and antibiotic use outside of the 12 to 21 month age range, and of improved toilet facility and toilet sharing at any age, were not statistically significant.

## Discussion

To our knowledge, this study describes the first application of LGCM to household-level risk factors associated with enteric infection and childhood linear growth. The study population is representative of an urban slum population in Bangladesh, where growth impairment is a particular challenge [20,32].

The decrease of LAZ across the 24 months is widely described in the literature [4,20]. A significant portion of growth impairment takes place between 6 and 24 months of age, when children are more mobile and may experience potential nutritional challenges and exposure to poor environmental conditions as breastfeeding is reduced and complementary foods are introduced [20,33].

As in previous studies, we have found a high prevalence of *Campylobacter* infection in this community. increasing throughout the first 24 months to reach a peak prevalence at 18 months of age (S1 Table). Again, supporting prior research, we found that *Campylobacter* infection has a negative association with linear growth; however, this study extends these observations by identifying the age intervals in the first 24 months of life where infection is most strongly associated with growth. Our analysis specifically identified that children between 12 and 21 months old are most likely to show a negative relationship between having had a *Campylobacter* episode 3 to 6 months previously and their age adjusted length (Fig 2).

We explored the impact of *Campylobacter* infection on age intervals instead of total attained height at the end of the study. This approach was applied to specifically examine the effect of infection on short term growth faltering and to identify the period most critical for the association between infection and growth impairment. A previous MAL-ED analysis across the 8 study sites described the second year of life as being the most critical period for *Campylobacter-*associated growth impairment. [8] Our findings provide further support that effect of exposure to potential *Campylobacter* pathogen reservoirs and resulting infection on growth may have been limited in the first year of child’s life, given that we did not find an association between infection and growth during this period. Interestingly, our analysis did not find an association between preceding infection and LAZ in the latest time interval, specifically at 24 months of age. This may suggest that the increasing and initial exposure to *Campylobacter* has the most significant effect on the growth trajectory of children. Most children may have already suffered an incident of *Campylobacter* infection by 18 to 21 months of age, with few new incidents of infection occurring after this period; however, further analyses would need to confirm this. In addition, children may develop protective immunity following early infection, which may explain the reduction in infection rates in addition to the high number of asymptomatic cases [34]. As pathogen data used for this analysis was collected from asymptomatic stool samples, this analysis further highlights the importance of asymptomatic *Campylobacter* infection on child growth. Additional analyses should also compare the effect of symptomatic stool samples.

Treatment of drinking water and increased duration of antibiotic use were important negative predictors for *Campylobacter* infection. Treatment of drinking water, as an important predictor for *Campylobacter* infection, may therefore be indirectly impacting positively upon growth. All participating households received their water from an improved piped water source as defined by World Health Organization Joint Monitoring Programme [35]; however, treatment of drinking water was positively associated with LAZ. Interestingly, the WASH Benefits Bangladesh study did not find a benefit in water treatment to the reduction of reported diarrhea. In addition, the integration of WASH interventions to nutrition did not have a benefit to child LAZ. [36,37]. This was in contrast to a previous intervention conducted in another community, which identified a 36% reduction in diarrhea in children from the water chlorination plus safe storage arm when compared to controls [37]. Another study reported that children from households with better conditions had higher LAZ scores compared with household with poorer hygienic conditions [38]. Our findings suggest that water treatment is having an effect on *Campylobacter* infection and indirectly on LAZ. Previous studies may not have found an effect of water treatment on enteric disease due to inadequately interrupting the pathogen-specific transmission pathways. The relationship between water treatment and *Campylobacter* infection identified in this analysis needs to be explored further.

Antibiotic use was prevalent in this study population. A previous study reported that the Bangladesh site had one of the highest rates of antibiotic use, second to the Pakistan site. They also found that antibiotic use in the prior month was associated with a reduced risk of *Campylobacter* detection in the stool [2]. While it was negatively associated with *Campylobacter* infection, there needs to be special consideration about its long-term effects beyond the first 24 months of age, given the current global health challenge of the increasing rate of antibiotic resistance and potential negative effects on the gut microbiota [39].

Previous research has suggested the exposure to chickens in the home as a risk factor for childhood impaired growth with LAZ; however, our analysis did not identify an association between chicken ownership and LAZ [8]. Interestingly, the Bangladesh site had one of the lowest rates of reported chicken ownership of all participating MAL-ED sites, with few households reporting the presence of chickens in the home (1.3%). However, reported chicken and fowl ownership may not accurately reflect children’s exposure to such zoonosis as *Campylobacter* since chickens from other households wander freely throughout the community and so may be a source of children’s exposure.

Increased maternal education in previous research has also as being associated with improved linear growth in young children [18,40,41]. In our preliminary models, maternal education was significantly associated with the rate of change of growth; however, after adjusting for other covariates in our final model, the relationship was no longer present. An analysis of the MAL-ED cohort did not find an association with breastfeeding practices and growth in the first 24 months, and commented that duration of exclusive breastfeeding was low among the participants [15]. Similarly, we did not identify an association between LAZ and *Campylobacter* among the Bangladesh participants.

Research on the implication of birth order on child growth is inconsistent. Several studies have reported that firstborns have lower birth weight but that they become significantly taller in later childhood and in adulthood, while others reported that they remained shorter [42,43]. In this analysis, increased birth order was positively associated with baseline LAZ of the children; however, it was not associated with the rate of change in LAZ. Maternal height was also associated with the intercept, or the birth length, of the participating children. However, they were not associated with the slope or rate of change of LAZ in the first 24 months of age.

### Limitations

Our small sample size limited our ability to include additional variables in this pathogen-specific analysis, given our focus on exploring the various household predictors for growth and *Campylobacter* infection. Dietary intake has widely been identified as having an effect on linear child growth and so future analysis of the extensive dietary intake data collected for the MAL-ED analysis should aim to identify key variables to incorporate in latent grow curve models. Our team is currently developing such models. Also, our analysis uses pathogen data collected from regularly-collected non-diarrheal samples; however, future analyses should also consider the pathogen information collected from diarrheal samples. Finally, the urban slum population may result in a generalizability limitation, with findings being specific to a densely populated urban settlement. However, given rapid rate of urbanization on the global scale, particularly in low- and middle-income countries, child health in urban settings is an increasingly important global health concern.

### Conclusion

Linear growth impairment in children is a complex public health challenge resulting from a variety of factors associated with poor socioeconomic conditions. At the same time, *Campylobacter* infection continues to be widely prevalent as part of these conditions, particularly in the Bangladesh MAL-ED population. To our knowledge, this is the first application of the latent growth curve modelling to explore *Campylobacter* infection, childhood linear growth, and the association with household factors. he negative association between *Campylobacter* infection and LAZ at 12, 15, 18 and 21 months of age reinforces the importance of addressing and controlling *Campylobacter* infection. Our household-level model also highlights the need to reduce the prevalence of infection by addressing the factors associated with it. While water sources in the households of participating children at the Bangladesh site were considered to be “improved” piped sources, treatment of drinking water was identified as being negatively associated with *Campylobacter* infection. Further examination using this modelling framework is needed to understand the relationship between water treatment and enteric infection.

## Supporting information

S1 Table Prevalence of enteric pathogens across age intervals. (PDF)
