# Correction: *Campylobacter* infection and household factors are associated with childhood growth in urban Bangladesh: An analysis of the MAL-ED study

**DOI:** 10.1371/journal.pntd.0009364

**Published:** 2021-04-20

**Authors:** The PLOS Neglected Tropical Diseases Staff

## Notice of republication

This article was republished on April 5, 2021, to correct errors in the author byline that were introduced during the typesetting process. The publisher apologizes for the errors. The second and fourth authors’ names were spelled incorrectly. The correct names are: Md. Ashraful Alam and Md. Ahshanul Haque. Please download this article again to view the correct version. The originally published, uncorrected article and the republished, corrected articles are provided here for reference.

## Supporting information

S1 File Originally published, uncorrected article. (PDF)

S2 File Republished, corrected article. (PDF)
