# Supplementary material for: Campylobacter infection and household factors are associated with childhood growth in urban Bangladesh: An analysis of the MAL-ED study
Source: PLoS Negl Trop Dis. 2020 May 14;14(5):e0008328. doi: 10.1371/journal.pntd.0008328 (PMC7252635; doi:10.1371/journal.pntd.0008328)
Supplement: S1 Table — (PDF) [file pntd.0008328.s001.pdf]

S1 Table. Prevalence of enteric pathogens across age intervals

Pathogen	1 month* N (%)	3 months N (%)	6 months N (%)	9 months N (%)	12 months N (%)	15 months N (%)	18 months N (%)	21 months N (%)	24 months N (%)
**Campylobacter**	20 (8.00)	41 (17.75)	41 (17.75)	106 (49.30)	124 (61.39)	120 (59.11)	145 (73.23)	142 (72.08)	133 (68.91)
**Cryptosporidium**	16 (6.40)	2 (0.87)	3 (1.38)	5 (2.33)	7(3.47)	4 (1.97)	10 (5.05)	9 (4.47)	4 (2.07)
**Astrovirus**	6 (2.40)	4 (1.73)	10 (4.61)	7 (3.26)	6 (2.97)	2 (0.99)	7 (3.54)	7 (3.55)	11 (5.70)
**EPEC**	2 (0.8)	3 (1.30)	15 (6.91)	28 (13.02)	17 (8.42)	14 (6.90)	8 (4.04)	5 (2.54)	4 (2.07)
**Giardia**	0	1 (0.43)	1 (0.46)	6 (2.79)	13 (6.44)	16 (7.88)	28 (14.14)	28 (14.21)	29 (15.03)
**Ascaris**	0	0	1 (0.46)	3 (1.40)	5 (2.48)	11 (5.42)	18 (9.09)	14 (7.11)	15 (7.77)
